# Phosphorus Over-Fertilization and Nutrient Misbalance of Irrigated Tomato Crops in Brazil

**DOI:** 10.3389/fpls.2017.00825

**Published:** 2017-05-19

**Authors:** Rodrigo H. D. Nowaki, Serge-Étienne Parent, Arthur B. Cecílio Filho, Danilo E. Rozane, Natália B. Meneses, Juliana A. dos Santos da Silva, William Natale, Léon E. Parent

**Affiliations:** ^1^Department of Plant Production, São Paulo State UniversityJaboticabal, Brazil; ^2^Department of Soils and Agri-Food Engineering, Université LavalQuebec, QC, Canada; ^3^Department of Agronomy, São Paulo State UniversityRegistro, Brazil; ^4^Department of Plant Science, Federal University of CearáFortaleza, Brazil

**Keywords:** *Solanum lycopersicum* L., compositional nutrient diagnosis, isometric log ratio, multivariate analysis, nutrient balance, critical range

## Abstract

Over the past 20 years, the use of center-pivot irrigation has increased tomato (*Solanum lycopersicum* L.) yields in Brazil from 42 Mg ha^−1^ to more than 80 Mg ha^−1^. In the absence of field trials to support fertilizer recommendations, substantial amounts of phosphorus (P) have been applied to crops. Additional P dosing has been based on an equilibrated nutrient P budget adjusted for low-P fertilizer-use efficiency in high-P fixing tropical soils. To document nutrient requirements and prevent over-fertilization, tissue samples and crop yield data can be acquired through crop surveys and fertilizer trials. Nevertheless, most tissue diagnostic methods pose numerical difficulties that can be avoided by using the nutrient balance concept. The objectives of this study were to model the response of irrigated tomato crops to P fertilization in low- and high-P soils and to provide tissue diagnostic models for high crop yield. Three P trials, arranged in a randomized block design with six P treatments (0–437 kg P ha^−1^) and three or four replications, were established on a low-P soil in 2013 and high-P soils in 2013 and 2014, totaling 66 plots in all. Together with crop yield data, 65 tissue samples were collected from tomato farms. We found no significant yield response to P fertilization, despite large differences in soil-test P (coefficient of variation, 24%). High- and low-yield classes (cutoff: 91 Mg fruits ha^−1^) were classified by balance models with 78–81% accuracy using logit and Cate–Nelson partitioning models. The critical Mahalanobis distance for the partition was 5.31. Tomato yields were apparently not limited by P but were limited by calcium. There was no evidence that P fertilization should differ between center-pivot-irrigated and rain-fed crops. Use of the P budget method to arrive at the P requirement for tomato crops proved to be fallacious, as several nutrients should be rebalanced in Brazilian tomato cropping systems.

## Introduction

Tropical soils are highly weathered and have low natural fertility, such that fertilization is required to achieve high crop yields (van Raij et al., 2001; van Raij, 2011). Phosphorus (P) is the nutrient that most limits crop productivity in tropical soils (Novais and Smyth, 1999; Salcedo, 2006), especially in Brazil (Roy et al., 2016). Recovery of P by crops is generally low, due to the strong sorption of P by iron (Fe) and aluminum (Al) oxyhydroxides (Fe_ox_ and Al_ox_, respectively) (Novais, 1977; Baligar and Bennett, 1986a,b). In São Paulo soils, amounts of Fe_ox_ and Al_ox_ extractable by acid ammonium oxalate solution range from 0.23 to 15.3 g Fe_ox_ kg^−1^ and from 0.2 to 9.92 g Al_ox_ kg^−1^, respectively (Leal et al., 2013). Brazilian latosols may adsorb more than 2 mg P cm^−3^ in the 0–20 cm layer, potentially building up large P reserves (Ker, 1995; Novais et al., 2007). Given that demand for P fertilizers has been rising by 2.2% annually (FAO, 2015), the easily accessible world reserves of phosphate rock may become exhausted within the next 50–100 years (Cordell et al., 2009). To conserve resources and avoid waste, use of P fertilizers must be tailored to meet actual crop requirements.

The tomato (*Solanum lycopersicum* L.) is the most economically important vegetable grown in Brazil, with 4 × 10^6^ metric tons produced annually (Anuário Brasileiro de Hortaliças, 2015). Fertilizer application accounts for 18% of tomato production costs (CEPEA-Centro de Estudos Avançados em Economia Aplicada, 2011). The P dosage determined by soil-test P data for rain-fed tomato crops in Brazil is 70–218 kg P ha^−1^ for dry cerrado, 40–250 kg P ha^−1^ for the mid-part of the São Francisco valley, and 44–175 kg P ha^−1^ for São Paulo state. In São Paulo state, an average tomato yield of 42 Mg ha^−1^ amounts to a nutrient removal through harvest of 13.6–19.4 kg P ha^−1^, depending on the cultivar (Barbosa, 1990; Trani et al., 1997). However, tomatoes grown under center-pivot irrigation now produce an average yield of 80 metric tons ha^−1^ (AIA, 2016). If we extrapolate from a nutrient removal through harvest of 16.5 kg P ha^−1^ for a baseline yield of 42 Mg ha^−1^, the amount of P removal needed to achieve a target yield of 100 Mg ha^−1^ is nearly 40 kg P ha^−1^, a difference of 23.5 kg P ha^−1^ compared to the baseline P removal. P fertilization could be inflated substantially depending on the ratio of P recovered by harvest, which ranges from 0.1 to 0.3 in tropical soils in general (Baligar and Bennett, 1986a,b) and from 0.3 to 0.6 in Brazilian states hosting substantive intensive agriculture (Roy et al., 2016). Such arbitrary decision-making regarding the additional P dosage can lead to increased production costs and potential eutrophication of surface waters (Haygart and Sharpley, 2000).

The ion diffusion coefficient, as the most important factor controlling phosphate ion availability, is influenced by the volumetric water content, which reduces the tortuosity of the diffusion path (Baligar and Bennett, 1986a). Liebscher's Law of the Optimum states that the contribution of any production factor (e.g., pH, weather, water, nutrients) that is in limited supply becomes greater the closer that other production factors are to their respective optima (De Wit, 1992). Thus, crop response to P fertilization varies not only with soil-test P and the availability of other nutrients and their interactions (Marschner, 1995; Wilkinson, 2000), but also with the ion diffusion coefficient, soil P buffering capacity, and water availability, factors that are important in controlling the transfer of phosphate ions to the roots at the solid–liquid interface (Barber, 1995). The concentration of a nutrient in plant tissues thus depends on the combined effects of soil, climate, and management factors on plant growth and nutrient acquisition (Munson and Nelson, 1990).

Plant tissue analysis is a useful complement to soil analysis. Results of tissue analysis can be interpreted by using different tissue diagnostic methods, such as ternary diagrams (Lagatu and Maume, 1934), critical nutrient ranges (Ulrich and Hills, 1967), multivariate analysis across concentration values (Holland, 1966), dual ratios (Güsewell, 2004), stoichiometric ratios (Ingestad, 1987), the Diagnosis and Recommendation Integrated System (Beaufils, 1973; Walworth and Sumner, 1987), and the Compositional Nutrient Diagnosis system (Parent and Dafir, 1992; Parent, 2011). The nutrient balance concept (Parent, 2011; Parent et al., 2012) is based on the isometric log ratio (ilr) transformation (Egozcue et al., 2003), which is the most appropriate method to conduct multivariate analysis on compositional data (Filzmoser et al., 2009), such as tissue analytical results. The ilr values account for nutrient interactions in plants by balancing the nutrients and can be integrated into a synthetic multivariate distance from the reference composition.

We hypothesized that supplemental P fertilization for irrigated tomato crops is unnecessary and wasteful because the approach used to calculate the additional P dosage neglects the beneficial effect of water on phosphate ion diffusion and neglects the mismanagement of other nutrients. The objectives of this paper were to: (1) determine the response of irrigated Brazilian tomato crops to P fertilization in low- and high-P-testing soils, (2) elaborate nutrient balance diagnosis for irrigated tomato crops, and (3) provide general guidelines to improve the fertilization of irrigated tomato crops in Brazil. The findings provide new insights into the fallacies of nutrient budgets to make fertilizer recommendations, into the development of nutrient balance models, and into the conduct of field experiments under to the law of the optimum.

## Materials and methods

### Data sets

Three tomato field experiments were conducted in São Paulo state between April and October in 2013 and 2014 in areas provided by Predilecta Foods Ltda. The soils were classified as red clay latosol (EMBRAPA, 2013) and averaged 520, 290, and 190 g kg^−1^ of clay, silt, and sand, respectively. The climatic regime was classified as Aw (tropical climate with a winter dry season), with a maximum temperature of 29.5°C, minimum temperature of 18.5°C, mean temperature of 24.2°C, and mean annual rainfall of 1,228 mm (INMET, 2014). The monthly temperature was comparable across sites and to the 10-year average. Precipitation was highly variable, especially in May and June (Table 1).

**Table 1:** **Climatic conditions at experimental sites in 2013 and 2014**.

**Month**	**Low-P and High-P soils, 2013**	**High-P soil, 2014**	**Low-P and High-P soils, 2013**	**High-P soil, 2014**
	**Mean temperature (°C)**		**Rainfall (mm)**	
April	23.2	24.6	60.9	66.6
May	21.3	21.9	144.0	9.3
June	21.5	22.2	50.6	0.2
July	20.1	21.0	5.3	18.4
August	27.8	29.3	6.8	0.0
September	28.9	31.0	65.8	71.5
October	28.6	32.4	89.1	22.0

*Source: INMET (2014)*.

The P trials were established on low-P soil in 2013 and high-P soils in 2013 and 2014. There were 66 plots across sites and years. Each plot consisted of three 6-m-long cultivation lines spaced 1.25 m from each other; plant spacing on the row was 0.25 m. In the high-P soil in 2013 (three replicates), treatment applications were 0, 65.5, 131, 196.5, 262, and 327.5 kg P ha^−1^. In the low-P and high-P soils in 2013 (three replicates) and 2014 (four replicates), respectively, treatment applications were 0, 87.3, 174.7, 262, 349.3, and 436.7 kg P ha^−1^. The P source was triple superphosphate (17.9% P) across all trials. All P treatments were combined with 30 kg nitrogen (N) ha^−1^ as urea and 60 kg potassium oxide (K_2_O) ha^−1^ as potassium chloride (KCl), which were applied in bands in open grooves before planting. Additional N and potassium (K) fertilizers were broadcast 30–35 days later, at 30 kg N ha^−1^ and 100 kg K_2_O ha^−1^, respectively. A further dosage of 30 kg N ha^−1^ was broadcast 20 days thereafter, to reach total dosages for the season of 90 kg N ha^−1^ and 160 kg K_2_O ha^−1^ (Trani et al., 1997).

Tomato seedlings (cv. Heinz 9553), grown in trays, were transplanted at the four-leaf stage. Irrigation was applied by center pivot. Crops were managed to control disease and pests as needed. Tomatoes reached maturity 110–120 days after planting. Crop productivity was determined by harvesting fruits from 20 plants per plot, from the mid-parts of the lines, neglecting 0.5 m at the end of each line. Commercial yield included red and partially red fruits. In addition, areas producing tomatoes for processing were surveyed at 65 farm locations in São Paulo state during the 2013 and 2014 cropping seasons. Fifteen cultivars were sampled in this survey: H9553, U2006, HM7883, Acangatá, AP529, AP533, HM7885, H9889, AP533, BA5630, Milagro, Umbopoq, AP-Seminis, HMX9553, and HMX7889. Together, the experimental and surveyed crops provided 131 samples for tissue diagnostic analysis.

### Soil analysis

Before establishing the plots, we obtained 20 subsamples of soil from the 0–0.20 m layer of each site, which we combined into 250-cm^3^ samples for chemical analysis (van Raij et al., 2001). P was extracted with Amberlite IRA-400 ion-exchange resin (20–50 mesh), quantified by colorimetry by the ascorbic acid method, and reported as mg dm^−3^. K, calcium (Ca), and magnesium (Mg) were extracted with Amberlite IRA-120 ion-exchange resin (20–50 mesh), quantified by flame photometry (K) or atomic absorption spectrophotometry (Ca, Mg), and reported in mmol_*c*_ dm^−3^. Copper (Cu), Fe, manganese (Mn), and zinc (Zn) were extracted with diethyltriaminepentaacetic acid and quantified by atomic absorption spectrophotometry. Boron (B) was extracted by the hot-water method and quantified by colorimetry. Total carbon was determined by dichromate oxidation (Abreu et al., 2006).

Dolomitic limestone (45–48% calcium oxide and 6–10% magnesium oxide) was applied to increase the base saturation of the cation exchange capacity (CEC) to 80% and the Mg concentration to at least 9 mmol_*c*_ dm^−3^ (Trani et al., 1997). The CEC was computed as the sum of the concentrations of K, Ca, Mg, and the exchangeable acidity. Exchangeable acidity was estimated by the Shoemaker–McLean–Pratt (SMP) buffer method and the following equation (Quaggio et al., 1985):

$$\left(\text{H}+\text{Al}\right)=10\text{exp}(7.76+1.053{\text{pH}}_{\text{SMP}}),R^{2}=0.98$$

The lime requirement (*LR*) was calculated as follows:

$$\text{LR}=[\text{CEC}({\text{B}}_{\text{2}}-{\text{B}}_{\text{1}})]\text{/}(\text{10}\times \text{TRNP})$$

where LR is lime requirement (tons ha^−1^), B_1_ is the base saturation of CEC, computed as 100 × (K^+^ + Ca^2+^ + Mg^2+^)/CEC on a molar basis, B_2_ is the targeted base saturation of CEC, and TRNP is the relative neutralizing power of the liming material according to Brazilian standards (van Raij et al., 1997).

### Tissue analysis

Ten first-mature leaves (fourth leaf from the top of each plant) were collected at the beginning of flowering in each plot of the experimental and survey sites (Fontes, 2000). Leaf samples were gently washed, oven-dried at 65°C, and ground to <1 mm in a Wiley mill. Ground leaves were digested in sulfuric acid to quantify Kjeldahl-N and in a mixture of nitric and perchloric acids to determine P, K, Mg, sulfur (S), copper (Cu), Fe, Mn, and Zn contents by plasma emission spectroscopy (ICP-OES). The B content was quantified by the azomethine-H colorimetric method, after tissue calcination (Malavolta et al., 1997).

### Compositional model

The compositional simplex *S*^*D*^, composed of *D* intrinsically multivariate and related tissue data, is described as follows (Aitchison, 1986):

$$S^{D}\text{​}=\text{​}\left\{x=\left[x_{1},x_{2},\ldots ,x_{D}\right]|x_{i}>0,\;i\text{​}=\text{​}1,2,\ldots ,D;\sum_{i=1}^{D}x_{i}\text{​}=\kappa \right\},$$

where $x_{D}=100\%-\;\sum_{i=1}^{D}x_{j},x_{i}$ is the *i*^*th*^ non-overlapping component of the whole, and κ represents closure to the measurement unit or scale. The filling value (*x*_*D*_) is computed as the difference between κ and the sum of quantified components.

Compositional data are related to each other such that if the value of one component increases, the value of at least one component must decrease. To avoid “resonance,” compositional data should be expressed as log ratios (Aitchison, 1986). Log ratio expressions are useful for conducting statistical analyses on compositional data because: (1) log(*A/B*) and log(*B/A*) ratios are reflective, i.e., log(*A/B*) = −log(*B/A*) (Beverly, 1987a,b); (2) the geometric mean is the most appropriate way to average ratios (Fleming and Wallace, 1986); (3) it is common to use a logarithmic scale when ratios are >10^4^ (Budhu, 2000); and (4) carefully arranged log ratios extract *D*–1 informative variables from *D*-part compositions (Aitchison and Greenacre, 2002).

Egozcue et al. (2003) and Egozcue and Pawlowsky-Glahn (2005) developed the ilr or orthonormal balance concept. The ilr value is computed as follows:

$$ilr_{j}=\sqrt{\frac{n_{j}^{+}n_{j}^{-}}{n_{j}^{+}+n_{j}^{-}}}ln\frac{g\left(c_{j}^{+}\right)}{g\left(c_{j}^{-}\right)},$$

where $n_{j}^{+}$ and $n_{j}^{-}$ are the numbers of components in the numerator and denominator, respectively; $g\left(c_{j}^{+}\right)$ and $g\left(c_{j}^{-}\right)$ are the geometric means across components of the numerator and denominator, respectively; and the coefficient $\sqrt{\frac{n_{j}^{+}n_{j}^{-}}{n_{j}^{+}+n_{j}^{-}}}$ allows the normalizing of orthogonal into orthonormal balances. Balances are presented as [components in denominator|components in numerator]. When the denominator of the log ratio contains higher concentration values, the balance becomes more negative and leans to the left, and inversely, as in algebra.

Although there are *D* × (*D*−1)/2^*D*^^−1^ possible combinations of *D*−1 orthonormal balances in a *D*-part composition (Pawlowsky-Glahn and Egozcue, 2011), some balances are more meaningful than others. *Ad hoc* balances may be elaborated from scientific literature, local knowledge, bi-plot analysis, management (e.g., fertilization, liming, foliar diseases), a specific hypothesis or theory, or randomly if the definition of the orthonormal space is not of concern, so long as balances remain orthogonal to each other. Because balances are orthonormal, any design returns the same results of multivariate analysis, which is the ultimate purpose of using the ilr (Filzmoser et al., 2009). The Mahalanobis distance between any specimen and a reference composition is computed as follows:

$$ℳ=\sqrt{{\left(ilr_{j}-ilr_{j}^{\text{*}}\right)}^{T}COV^{-1}\left(ilr_{j}-ilr_{j}^{\text{*}}\right)}$$

where $ilr_{j}^{*}$ is the mean and COV is the covariance matrix of the reference population.

The balance design used in this paper is described by the sequential binary partition in Table 2. For 11 nutrients, 10 balances were selected to represent nutrient dilution in the tissue mass (ilr11), fungicide, fertilizer, and lime-related nutrients, and the magnitude of nutrient uptake, as well as specific nutrient uptake rate, functions, and relationships (Malavolta, 2006). The order of nutrient uptake in the tomato plant is K > N > Ca > S > Mg = P (Fayad et al., 2002). P and Mg are involved in photosynthesis and cellular energetics in general. Ca and B are involved in cell-wall and membrane stability (Marschner, 1995). N and S are involved in protein synthesis (Parent et al., 2013). K acts in the regulation of stomatal opening, ion transport, and enzyme activation (Taiz and Zeiger, 2004). Although the sole source of Fe is the soil, fungicides may supply other cationic micronutrients, including Cu, Zn, and Mn.

**Table 2:** **Sequential binary partitions of tomato leaf analytical data**.

**ilr**	**Balance**	**K**	**Mg**	**N**	**P**	**S**	**Ca**	**B**	**Fe**	**Cu**	**Zn**	**Mn**	**x_*D*_**	***r***	***s***
1	[Mg | K]	1	−1	0	0	0	0	0	0	0	0	0	0	1	1
2	[P | N]	0	0	1	−1	0	0	0	0	0	0	0	0	1	1
3	[N, P | K, Mg]	1	1	−1	−1	0	0	0	0	0	0	0	0	2	2
4	[S | K,Mg, N, P]	1	1	1	1	−1	0	0	0	0	0	0	0	4	1
5	[B | Ca]	0	0	0	0	0	1	−1	0	0	0	0	0	1	1
6	[B, Ca | K, Mg, N, P, S]	1	1	1	1	1	−1	−1	0	0	0	0	0	5	2
7	[Fe, Cu, Zn, Mn | K, Mg, N, P, S, Ca, B]	1	1	1	1	1	1	1	−1	−1	−1	−1	0	7	4
8	[Mn | Zn]	0	0	0	0	0	0	0	0	0	1	−1	0	1	1
9	[Mn, Zn | Cu]	0	0	0	0	0	0	0	0	1	−1	−1	0	1	2
10	[Mn, Zn, Cu | Fe]	0	0	0	0	0	0	0	1	−1	−1	−1	0	1	3
11	[*x*_*D*_ | Fe, Cu, Zn, Mn, K, Mg, N, P, S, Ca, B]	1	1	1	1	1	1	1	1	1	1	1	−1	11	1

*ilr, isometric log ratio coordinate (orthonormal balance); x_D_, filling value; r and s: numbers of components in plus (+) and minus (−) groups, respectively*.

### Statistical methods

We performed statistical computations using the R language (R Core Team, 2017) and analyzed the P experiment as a mixed model using the R nlme package (Pinheiro et al., 2017), with P dose, soil P saturation (low or high P), and their interaction as fixed effects and trials and repetitions as random effects. Nutrient compositions were transformed to balances by using the R compositions package (van den Boogaart et al., 2014). The effect of tissue composition on yield was tested by using a mixed model with the nlme package. A binomial logit linear model across nutrient balances was used to classify data with a yield cutoff of 91 Mg ha^−1^. The logit model was computed as follows:

$$logit\left(p\right)=ln\left(\frac{p}{1-p}\right),\;0\leq p\leq 1$$

The probability of exceeding the yield target of 91 Mg ha^−1^ was computed as follows:

$$\text{p}=\frac{e^{y}}{1+e^{y}}$$

where *y* = *logit*(*p*). A logit value of zero corresponded to a probability of 0.5 of obtaining a high yield. The logit model was computed from a training dataset (75% of the dataset) and validated with a testing dataset (remainder of the dataset).

Group classification about a critical Mahalanobis distance was conducted by the Cate–Nelson partitioning procedure across the whole data set (Parent et al., 2013). The logit and Cate–Nelson models returned the following classification, as commonly reported in clinical studies (Parent et al., 2013): (1) true negative (TN) specimens (high yield, above logit of 0 or the critical Mahalanobis distance); (2) true positive (TP) specimens (low yield, below logit of 0 or the critical Mahalanobis distance); (3) false positive (FP) specimens (high yield, below logit of 0 or the critical Mahalanobis distance; type I error); and (4) false negative (FN) specimens (low yield, above logit of 0 or the critical Mahalanobis distance; type II error). Performance of the classification models was measured based on accuracy (i.e., probability that an observation could be correctly identified as balanced or imbalanced) and computed as (TN+TP)/(TN+FN+TP+FP). We also computed specificity (TN/TN+FP), sensitivity (TP/FN+TP), negative prediction value [NPV = (TN/TN+FN)], and positive predictive value [PPV + (TP/FP+TP)].

To derive concentration ranges at high- and low-yield levels, we ran 10^5^ Monte Carlo simulations across ilr confidence intervals (*P* < 0.05) of the TN and TP specimens (De Souza et al., 2016). Simulated ilr values were back-transformed into concentration values. Minimum and maximum concentrations delineated the univariate TN and TP nutrient ranges. However, the ilr and concentration ranges should be interpreted with care and for comparison purposes only, due to the joint distribution and multivariate nature of compositional data.

## Results and discussion

### Soil properties

Soil chemical properties varied widely among experimental sites (Table 3). Liming was sufficient to reach a pH value of at least 5.5 (Van Lierop, 1990) and to neutralize any toxic exchangeable Al across plots. Exchangeable Al was not detected in the low-P site in 2013. For the high-P soil in 2014, base saturation exceeded 80% (Table 3). Although other soils received dolomitic lime in sufficient amounts to reach 80% base saturation, the soil reaction to lime remained incomplete.

**Table 3:** **Properties of red clay latosols at experimental sites in 2013 and 2014**.

**Property**	**High-P soil, 2013**	**High-P soil, 2014**	**Low-P soil, 2013**
pH_CaCl2_	5.6	5.9	4.5
	**g dm**^−3^		
Organic matter	29	15	26
	**mg dm**^−3^		
Resin P	116	122	8
	**mmol_c_ dm^−3^**		
Exchangeable K	3.5	4.2	1.1
Exchangeable Ca	28	70	8
Exchangeable Mg	6	22	6
Sum of cationic bases	37.5	96.2	15.1
Cation exchange capacity (CEC)	66	116	51
Exchangeable acidity	29	20	36
Exchangeable Al	0	0	0
	**%**		
Saturation of CEC	30	83	57

Soil-test P data in Table 3 are reported as resin P, rather than Mehlich-1 P as found in previous studies in Brazil. Although the resin and Mehlich-1 methods are not directly comparable, their soil fertility groupings may be compared. For rain-fed crops, soil-test Mehlich-1 P is classified into fertility groups (mg Mehlich-1 P dm^−3^) as follows: Mehlich-1 *P* < 5 (low P), 6–10 (medium P), 11–20 (high P), and 21–40 (very high P), in the São Francisco river valley (Barbosa, 1990), compared to Mehlich-1 *P* < 10, 10–30, and > 30 in the Brazilian Cerrado (Câmara et al., 1985). In São Paulo state, soil-test *P*-values of <25, 26–60, and > 60 mg of resin P dm^−3^ are considered to be low (below optimal level), medium (close to optimal), and high (above optimal level), respectively (Trani et al., 1997). In lower-P soil, the resin P concentration was 8 mg dm^−3^, within the low soil-test range for which a significant yield response to P fertilization was expected. High-P soils (116–122 mg of resin P dm^−3^) showed extremely high soil-test *P*-values for which no yield response was expected.

### Response of tomato crops to added P

**Figure 1:**
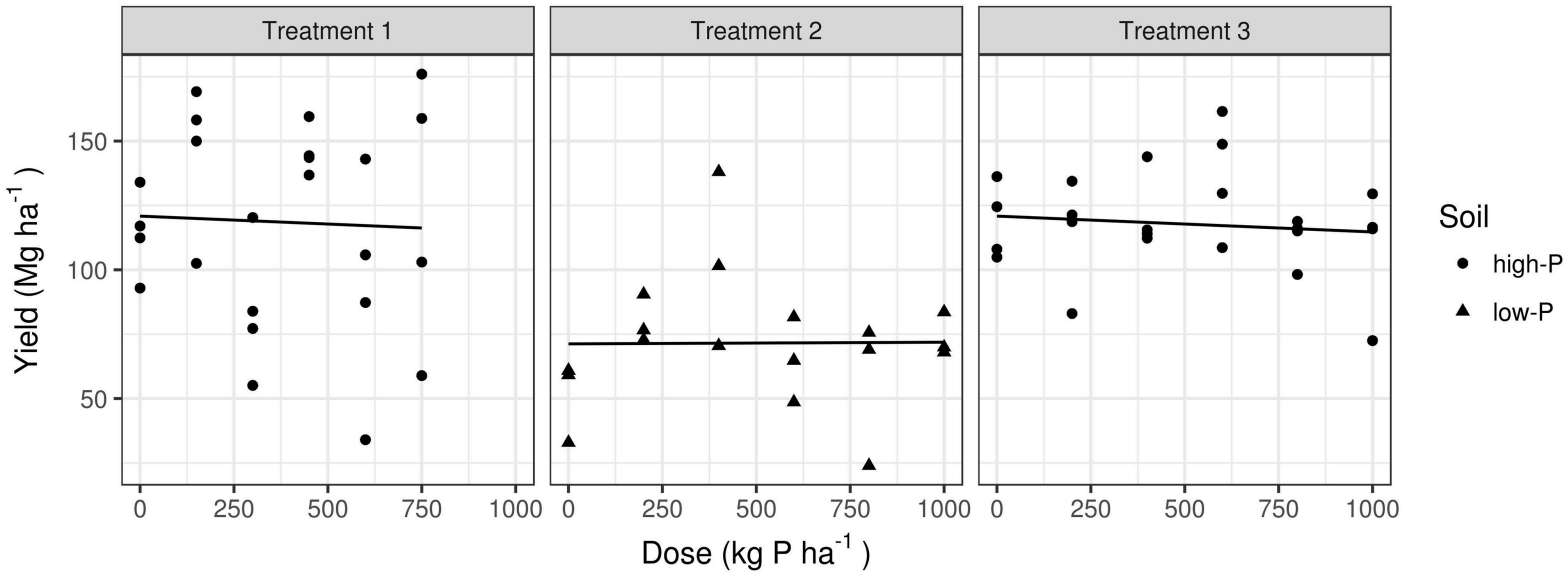
**Linear mixed models showing yield responses to P dosage in low-P and high-P soils**.

Results from the three experimental sites are presented in Table 4. At the plot level in high-P soils, tomato yield averaged 118 Mg fruits ha^−1^ (range: 34–176 Mg fruits ha^−1^) in 2013, compared to 119 Mg fruits ha^−1^ (range: 73–162 Mg fruits ha^−1^) in 2014. In the lower-P soil, yield averaged 72 Mg fruits ha^−1^ (range: 24–138 Mg fruits ha^−1^) in 2013. In these experiments, neither the P dose nor soil-test P showed any significant effect on yield. Linear responses of tomato fruit yield to P fertilization and soil P saturation are shown in Figure 1. The slope of the dose was 0.00063 Mg fruits ha^−1^ per kg P ha^−1^ (*P* = 0.975). The effect of high-P compared to low-P averaged 49.6 Mg fruits ha^−1^ (*P* = 0.18). For the interaction, the slope of the P dose increased marginally by 0.007 in the low-P soil compared to high-P soil (*P* = 0.78). Standard deviations computed at the trial and replication levels were 2 × 10^−7^ and 5 × 10^−8^ Mg ha^−1^, respectively.

**Table 4:** **Tomato fruit yields in response to added P at the three experimental sites**.

**Rate**	**Low-P soil, 2013**	**High-P soil, 2014**	**Rate**	**High-P soil, 2013**
**kg P ha^−1^**	**Mg fruits ha^−1^**		**kg P ha^−1^**	**Mg fruits ha^−1^**
0	51.0^†^	118.4^†^	0	114.1^†^
87.3	80.0	114.4	65.5	145.0
174.7	103.3	121.4	131	84.1
262	65.0	137.2	196.5	146.1
349.3	56.2	112.0	262	92.5
436.7	73.8	108.6	327.5	124.2

† *No significant difference was found between P doses on the same column at P = 0.05 (coefficient of variation = 24%)*.

In rain-fed tomato cropping systems, Baumgartner et al. (1983) observed a significant yield response to P additions in a red latosol containing 8–14 mg Mehlich-1 P dm^−3^ (low-to-medium soil-test P). In tomato crops grown in a São Paulo sandy soil (pH_water_ = 5.8) containing 1 mg Mehlich-1 P dm^−3^ (low soil-test P), Barbosa (1990) obtained a significant yield response to P fertilization. Yield increased from 5.4 Mg fruits ha^−1^ in the control to 48.4 Mg fruits ha^−1^ with application of 131 kg P ha^−1^ or 49.1 Mg fruits ha^−1^ with application of 196 kg P ha^−1^. Similarly, for rain-fed tomato crops in the São Francisco river valley, De Faria et al. (1999) reported maximum economical yields 53 and 69 Mg fruits ha^−1^ in response to additions of 62 and 79 kg P ha^−1^ in soil containing 1 and 8 mg Mehlich-1 P dm^−3^ (low soil-test P), respectively. In contrast, crops grown in soils containing 14 mg Mehlich-1 P dm^−3^ (medium soil-test P) and 33–40 mg Mehlich-1 P dm^−3^ (high soil-test P) were not responsive to P fertilization.

Therefore, there is no indication that the P dosage should exceed the current Brazilian P recommendation standards based on soil-test P, even though much higher tomato yields are obtained with center-pivot irrigation. Supplemental application of P based on the increase in yield driven by irrigation represents an economic loss and waste of resources for growers and leads to P accumulation in the soil, increasing the risk of eutrophication of surface waters.

### Limiting nutrients at experimental sites

The binomial logit model performed well in diagnosis, classifying low and high yielders (delimited at 91 Mg fruits ha^−1^) with an accuracy of 78% for the training and testing datasets. Coefficients of the logit model and their *P*-values are presented in Table 5. To compute the logit, coefficients were multiplied by the ilr values, and the result was added to the intercept. If the result was >0, then the model was considered to predict a probability of >50% of attaining a high yield (>91 Mg fruits ha^−1^).

**Table 5:** **Coefficient of predictive binomial logit model elaborated from testing data set**.

**Balance**	**Coefficient**	***p*-value**
Intercept	32.8614	0.08
[Mg | K]	1.4279	0.38
[P | N]	−0.9342	0.64
[N, P | K, Mg]	−0.7887	0.60
[S | K, N, P, Mg]	−0.9020	0.39
[B | Ca]	0.5345	0.50
[B, Ca | S, K, N, P, Mg]	−0.8864	0.45
[Fe, Mn, Zn, Cu | S, K, N, P, Mg, B, Ca]	1.7629	0.04
[Mn | Zn]	−0.9797	0.13
[Mn, Zn | Cu]	−0.5055	0.31
[Mn, Zn, Cu | Fe]	−0.6991	0.25
[x_*D*_ | Fe, S, Mn, Zn, Cu, K, N, P, Mg, B, Ca]	6.7078	0.05

**Figure 2:**
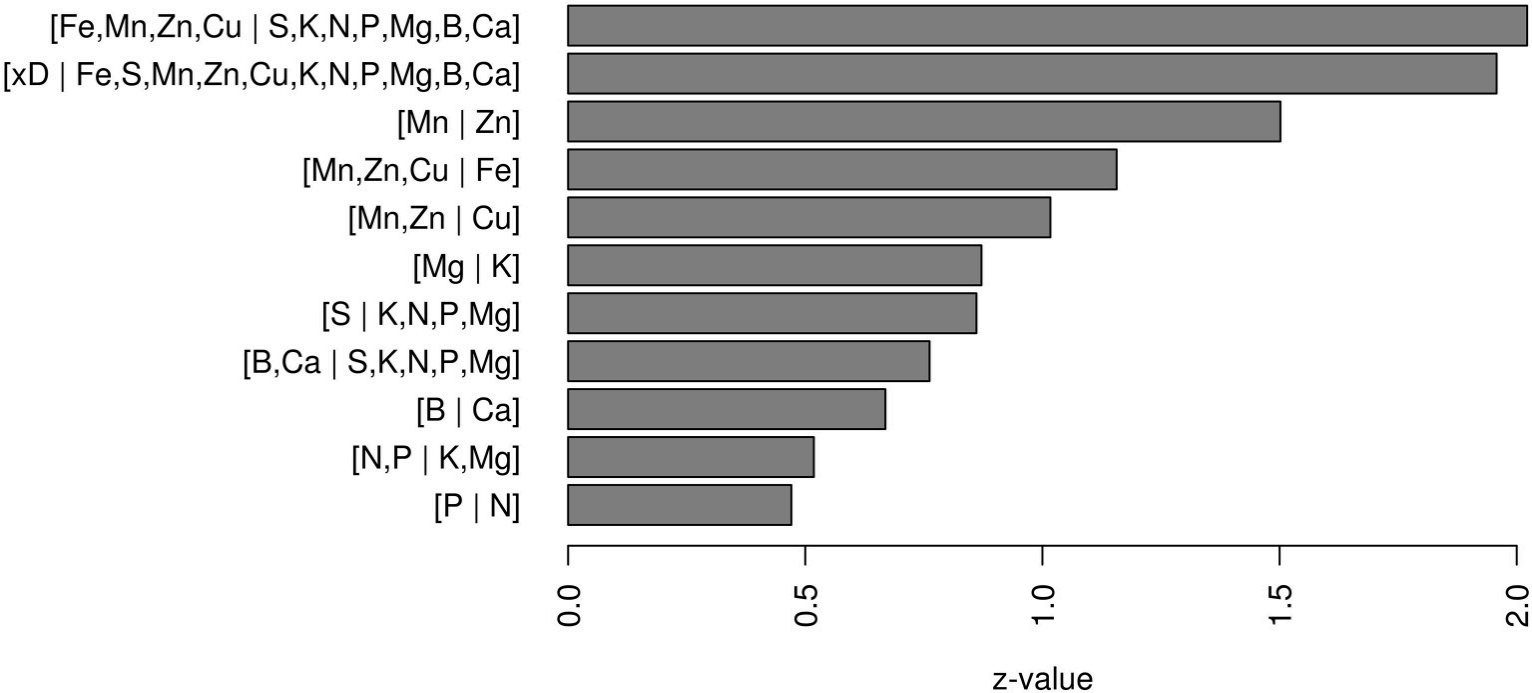
**Relative importance of *z*-values of balances in the logit model**.

The *z*-values showed the relative weights of nutrient balances in the logit model (Figure 2). The model detected a significant positive effect of the [Fe, Mn, Zn, Cu | S, K, N, P, Mg, B, Ca] balance. The significant and positive effect of the [x_*D*_| Fe, S, Mn, Zn, Cu, K, N, P, Mg, B, Ca] balance indicated that the probability to obtain high yield increased with increasing proportions of nutrients.

**Figure 3:**
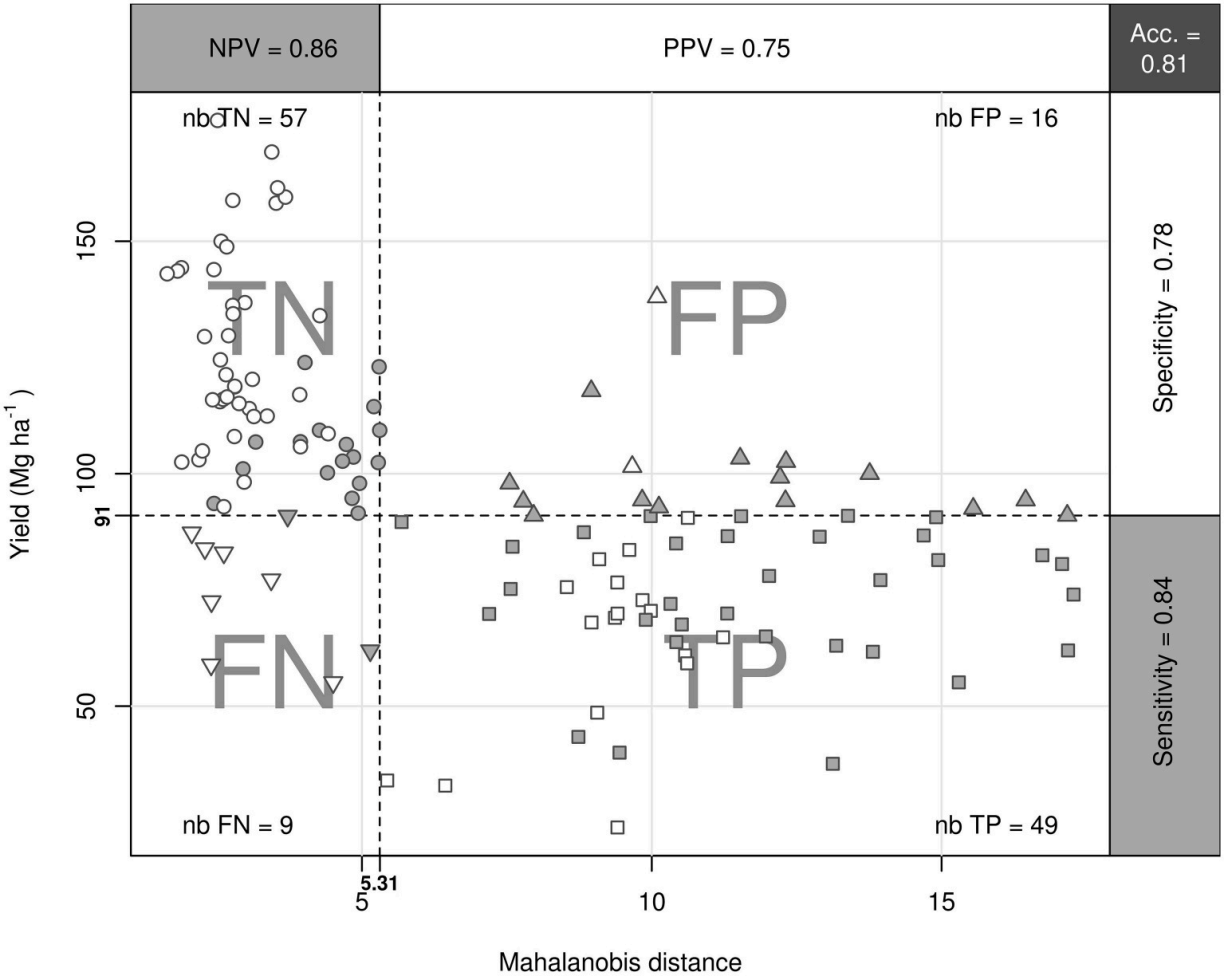
**Cate–Nelson partitioning of tomato dataset**. Data were partitioned into true negative (TN), false negative (FN), false positive (FP), and true positive (TP) specimens (nb = number). Empty and filled dots represent experimental data and survey data, respectively. Performance indices are the negative predictive value (NPV), positive predictive value (PPV), accuracy (Acc), specificity, and sensitivity.

The logit model returned 64 TN, 9 FN, 38 TP, and 20 FP specimens, corresponding to 78% accuracy, 76% specificity, 81% sensitivity, 87% NPV, and 66% PPV across the training and testing datasets. The Cate–Nelson partitioning method returned 81% accuracy, 78% specificity, 84% sensitivity, 86% NPV, and 75% PPV, for a cut-off yield of 91 Mg fruits ha^−1^ and a critical Mahalanobis distance of 5.31 between balanced and misbalanced specimens (Figure 3). FP specimens which showed high yield but misbalanced nutrition indicated possible cases of luxury consumption and leaf contamination. By contrast, FN specimens which showed low yield but balanced nutrition indicated that factors other than nutrition may have affected yield. Specimens in the TN and TP quadrants were almost evenly distributed among growers' fields and P trials. The logit and Cate–Nelson partitioning models provided complementary information on the relationship between plant nutrition and yield.

### Comparing nutrient balances of true-balanced and true-misbalanced specimens

**Figure 4:**
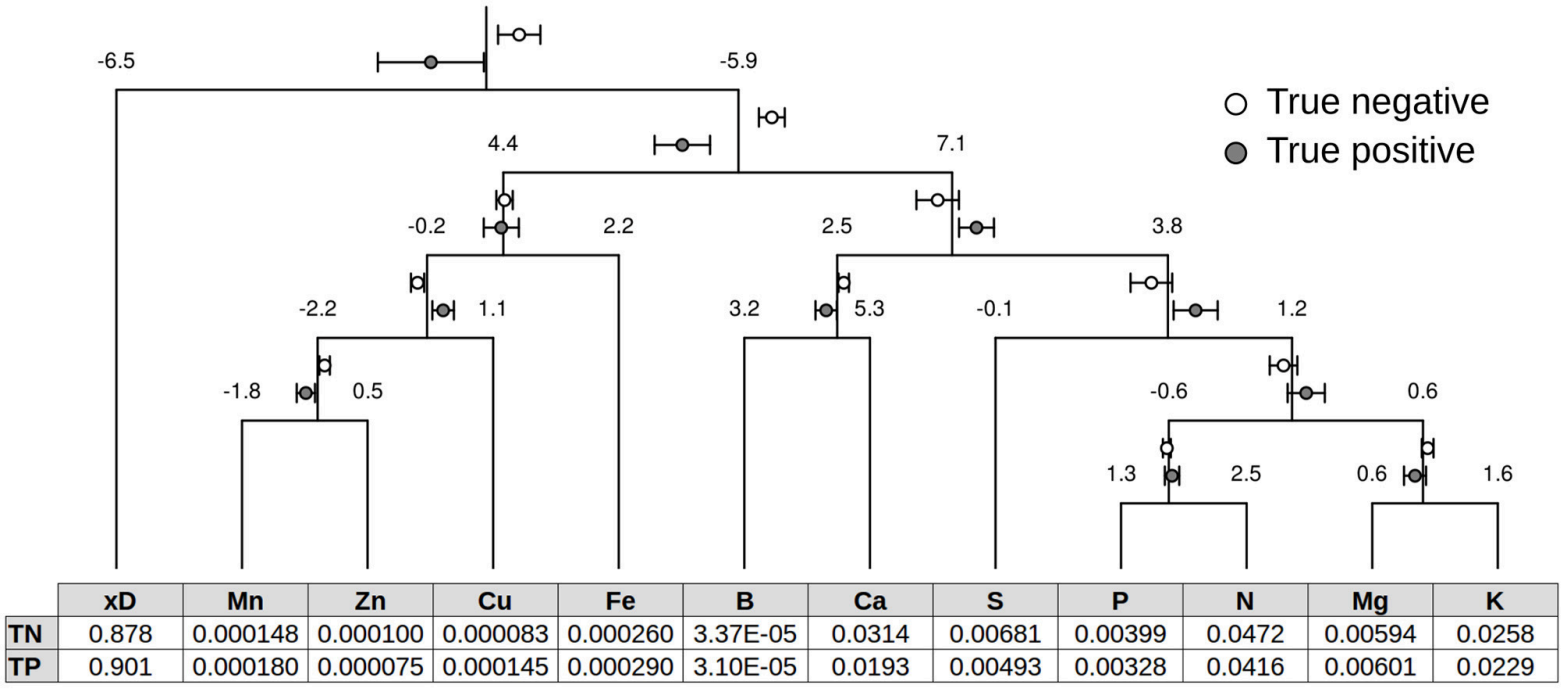
**Compositional dendrogram representing the balance scheme**. Confidence intervals (*P* = 0.05) are shown around the ilr means of true negative (TN) specimens at fulcrums. Concentration centroids back-transformed from ilr means of TN and true positive (TP) specimens are presented in buckets.

Figure 4 shows a compositional dendrogram with confidence intervals about ilr means of the TN and TP specimens. Nine balance means differed significantly at the 0.05 level between the TN and TP specimens of the Cate–Nelson partition. The [x_*D*_|nutrient] balance leaned significantly (*P* = 4.5 × 10^−4^) to the nutrient side for TN compared to TP specimens, indicating larger proportions of nutrients in the TN specimens. The [Fe, Mn, Zn, Cu|S, K, N, P, Mg, B, Ca] balance was significantly (*P* = 1.7 × 10^−9^) higher in TN compared to TP specimens. The [B, Ca|S, K, N, P, Mg] balance significantly (*P* = 0.013) leaned to the B and Ca side of TN compared to TP specimens. The [B|Ca] balance leaned significantly (*P* = 0.001) toward the Ca side of the TN specimens. The [S|K, N, P, Mg] balance of TN specimens leaned significantly (*P* = 0.007) toward the S side compared to TP specimens. The [N, P|K, Mg] balance significantly (*P* = 0.05) leaned toward the N and P side of the TN compared to TP specimens. For the [Mg|K] balance, the TN specimens tended to lean significantly (*P* = 0.03) toward the K side. In the cationic micronutrients subsystem, the [Mn, Zn|Cu] balance of TP specimens was significantly (*P* = 3.9 × 10^−5^) higher and the [Mn|Zn] balance was significantly (*P* = 1.9 × 10^−4^) lower compared to the TN specimens. No significant differences between TN and TP specimens were found in the [P|N] and [Mn, Zn, Cu|Fe] balances (*P* = 0.18 and 0.70, respectively).

Diagnoses using these ilr ranges are presented as a collection of separate variables. These results should be interpreted with care because the balances are distributed in a multidimensional space and weighted by a covariance matrix. The joint normal distribution of balances can be defined by a hyper-ellipsoid embedded within a hyper-cube of confidence intervals. Using the R calculator, we found that 0.092% of the volume of an ideal 11-dimensional hyper-cube was occupied by an ideal 11-dimensional hyper-sphere. Depending on the covariance matrix, the size of the critical hyper-ellipsoid shrunk toward ilr centroids as more balances were included.

The concentration centroids are reported in Figure 4. For comparison with other studies, we simulated concentration ranges (Table 5) using the minimum and maximum values of back-transformed randomly distributed TN balances (*P* = 0.05) and Monte Carlo simulations across those ranges. We found that 90% of the randomly recombined concentration values located within the 12 concentration ranges were outside the hyper-cube defined by the 11-ilr confidence intervals (*P* = 0.05) of TN specimens. Several centroids of the Trani et al. (1997) ranges for tomato crops in Brazil, such as P, K, B, and Zn (Table 6), differed markedly from those shown in Figure 4. This discrepancy likely occurred because (1) only TN specimens (no FP specimens) were used to compute concentration ranges, and (2) concentration ranges become narrower with increasing information about nutrient compositions and interactions (number of balances).

**Table 6:** **Confidence intervals for true negative (TN) and true positive (TP) tomato crops about the isometric log ratio (ilr) means, back-transformed to concentration values**.

**Nutrient**	**TN specimens**		**TP specimens**		^*^Reference	
	**LL**	**UL**	**LL**	**UL**	**LL**	**UL**
	**g kg^−1^**					
N	41.0	54.2	33.3	51.9	40	60
P	3.47	4.59	2.62	4.09	4	8
K	22.4	29.8	18.1	29.0	30	50
Ca	26.6	37.1	14.6	25.4	14	40
Mg	5.12	6.85	4.75	7.60	4	8
S	5.82	7.96	3.99	6.09	3	10
*x*_*D*_	869	887	884	915	−	−
	**mg kg^−1^**					
B	28.5	39.9	23.3	41.2	30	100
Cu	67.3	102	98.0	216	5	15
Fe	221	306	203	414	100	300
Mn	119	182	118	270	50	250
Zn	80.7	124	49.9	114	30	100

*LL, UL: lower and upper limits, respectively*.

* *Current nutrient concentration standards in Brazil (Trani et al., 1997)*.

Foliar P was not limiting at experimental sites because P trials showed no significant crop response to added P. However, the large proportion of misbalanced specimens (TP + FP) in Figure 4 indicated that the levels of non-P nutrients and the soil properties (e.g., low pH) of the lower-P soil were not optimal. By identifying nutrient misbalances (i.e., cases where Mahalanobis distance > 5.31), we could ascertain the yield limitations due to nutrients other than those being varied. We found relatively low Ca, S, and Zn levels in TP specimens. The Cu and Mn levels were presumably high due to the large fungicide applications by growers or foliar sampling close to fungicide spraying.

In fertilization trials, it is often assumed that factors other than those being varied are equal or set at sufficient or optimal levels to maximize crop response to the tested factors (De Wit, 1992). Foliar compositions are instrumental in identifying possible sources of nutrient misbalance at experimental sites. The diagnosis is first conducted in the balance domain against the critical Mahalanobis distance (in this study, 5.31). Thereafter, buckets are filled through fertilization or emptied by nutrient removal through harvest until the proper balance is reached. Adding Ca as lime material to fill the Ca bucket of TP specimens in Figure 4 will increase soil pH, hence potentially increasing soil availability of other nutrients.

## Conclusion

Center-pivot-irrigated tomato crops grown under conditions of low to very high soil-test P showed no significant response to P fertilization. This finding refutes the practice of P fertilization based on the increase in the amount of P removed through harvest and the proportion of fertilizer P that could be recovered by the crop. Tissue P showed a nonlimiting P supply regardless of the soil-test *P*-value and P treatment, confirming that there was little response to P fertilization for center-pivot-irrigated tomato plants grown at a soil-test *P*-value higher than 8 mg resin P dm^−3^. Using a logit model, we computed the probability of attaining a high yield level from tissue compositions arranged into balances with an accuracy of 78%. We also used another balance diagnostic model, which relied on nutrient centroids and a covariance matrix rather than inaccurate ranges of nutrients diagnosed separately, and on a critical multivariate distance, which had an accuracy of 81%. The critical Mahalanobis distance was 5.31 across 11 nutrient balances for tomato crops.

In Brazilian tropical soils, several nutrients could be rebalanced to attain fruit yields above 91 Mg fruits ha^−1^. Brazilian tomato growers could increase crop profitability by avoiding P overfertilization and supplying Ca and other imbalanced nutrients via proper fertilization and liming practices. Nutrient balance diagnosis could be instrumental in site selection for conducting fertilization trials by identifying potential yield-limitations of nutrients other than the ones being varied.

## Author contributions

Conceived and designed the experiments: RN, AC, and DR. Performed the experiments: RN, NM, JS. Analyzed the data: RN, SP, and LP. Wrote the paper: RN, SP, AC, WN, and LP.

### Conflict of interest statement

The authors declare that the research was conducted in the absence of any commercial or financial relationships that could be construed as a potential conflict of interest.
